# Supplementary material for: Central medial thalamic nucleus dynamically participates in acute itch sensation and chronic itch-induced anxiety-like behavior in male mice
Source: Nat Commun. 2023 May 3;14:2539. doi: 10.1038/s41467-023-38264-4 (PMC10156671; doi:10.1038/s41467-023-38264-4)
Supplement: Supplementary file 1 — Supplementary Information [file 41467_2023_38264_MOESM1_ESM.pdf]

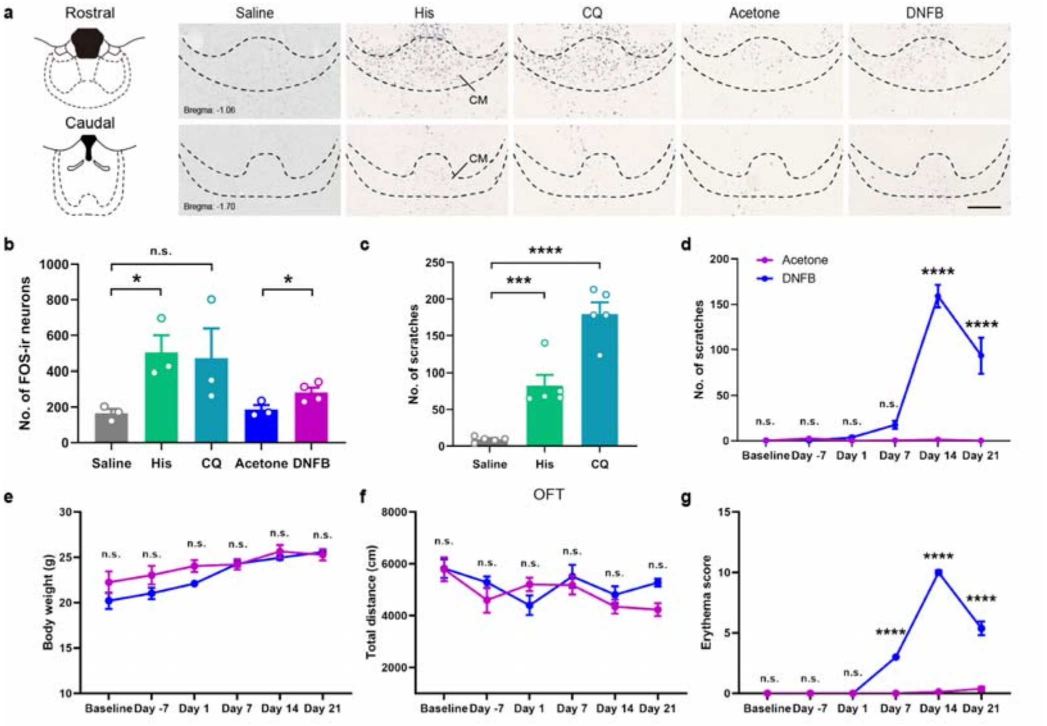

Supplementary Fig. 1 Effective acute and chronic itch models increased FOS expression in the CM. a, The rostral-caudal distribution of FOS-immunoreactive (ir) neurons in the CM. Scale bar=200 µm. Black dashed lines were used to lineate the boundaries of the CM. **b**, The number of FOS-ir neurons in both acute (His- and CQ-injected groups with saline as control; His vs. Saline: P=0.0266, CQ vs. Saline: P=0.1434; *n*=3 per group) and chronic itch models (DNFB-applied group with Acetone as control; DNFB vs. Ace: P=0.0499, *n*=3 for Ace group, *n*=4 for DNFB group) in quantitative analysis. **c**, Both His and CQ induced an increase in the number of scratches (His vs. Saline: P=0.001, CQ vs Saline: P < 0.0001; *n*=5 per group). **d**, DNFB-induced chronic itch model elevated the number of scratches, which peaked at day 14 (interaction: F(5,72)=34.38, P < 0.0001; Ace: *n*=6; DNFB: *n*=8). **e**, Body weight of mice in Acetone and DNFB groups (interaction: F(5,78)=1.184, P=0.3249; Ace: *n*=8; DNFB: *n*=7). **f**, Locomotor activity of mice applied with Acetone and DNFB (interaction: F(5,78)=1.722, P=0.1393; Ace: *n*=7; DNFB: *n*=8); **g**, The skin erythema score of mice when conducting Acetone and DNFB models (interaction: F(5,84)=232.5, P < 0.0001; Ace: *n*=7; DNFB: *n*=8). n.s.: no significance, *P < 0.05, ***P < 0.001, ****P < 0.0001. Data are presented as mean ± S.E.M. Two-tailed, unpaired, Student’s t-test for **b-c**, and two-way ANOVA with Sidak's multiple comparisons test for **d-g**. CQ: chloroquine; His: histamine; OFT:

open field test.

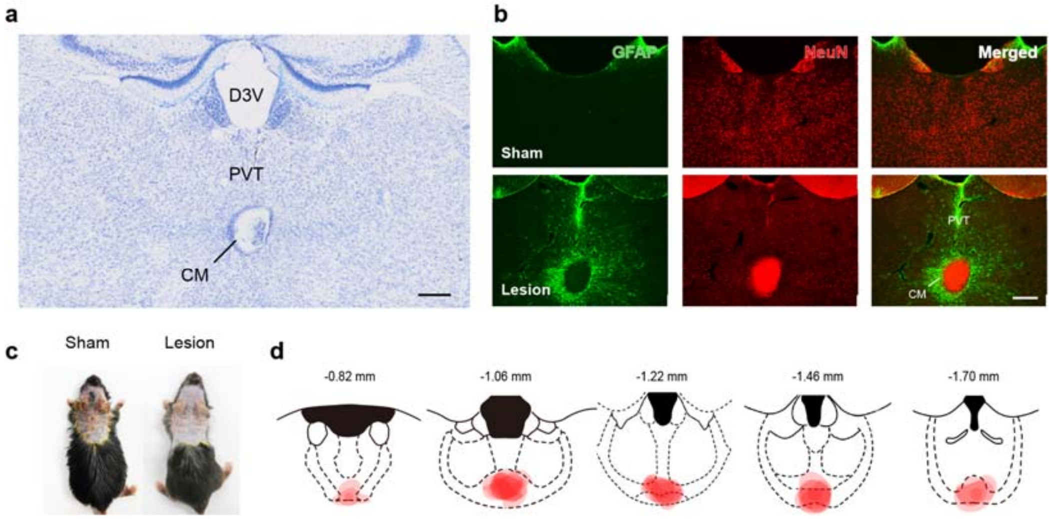

Supplementary Fig. 2 Morphological evidence of CM electrolytic lesion. **a**, The current spot induced by electrolytic lesion in CM is delineated with Nissl staining. Scale bar=100 µm. **b**, Double immunofluorescence staining of GFAP (green) and NeuN (Red) in Sham and Lesion groups (repeated in 25 successfully lesioned mice). Scale bar=100 µm. **c**, Representative photos of skin lesion in chronic itch model from Sham and CM lesion groups. **d**, Rostral-caudal distribution of the lesion range (red shadow) in mice. D3V: dorsal 3rd ventricle; CM: central medial thalamic nucleus; GFAP: Glial fibrillary acidic protein; PVT: paraventricular thalamic nucleus.

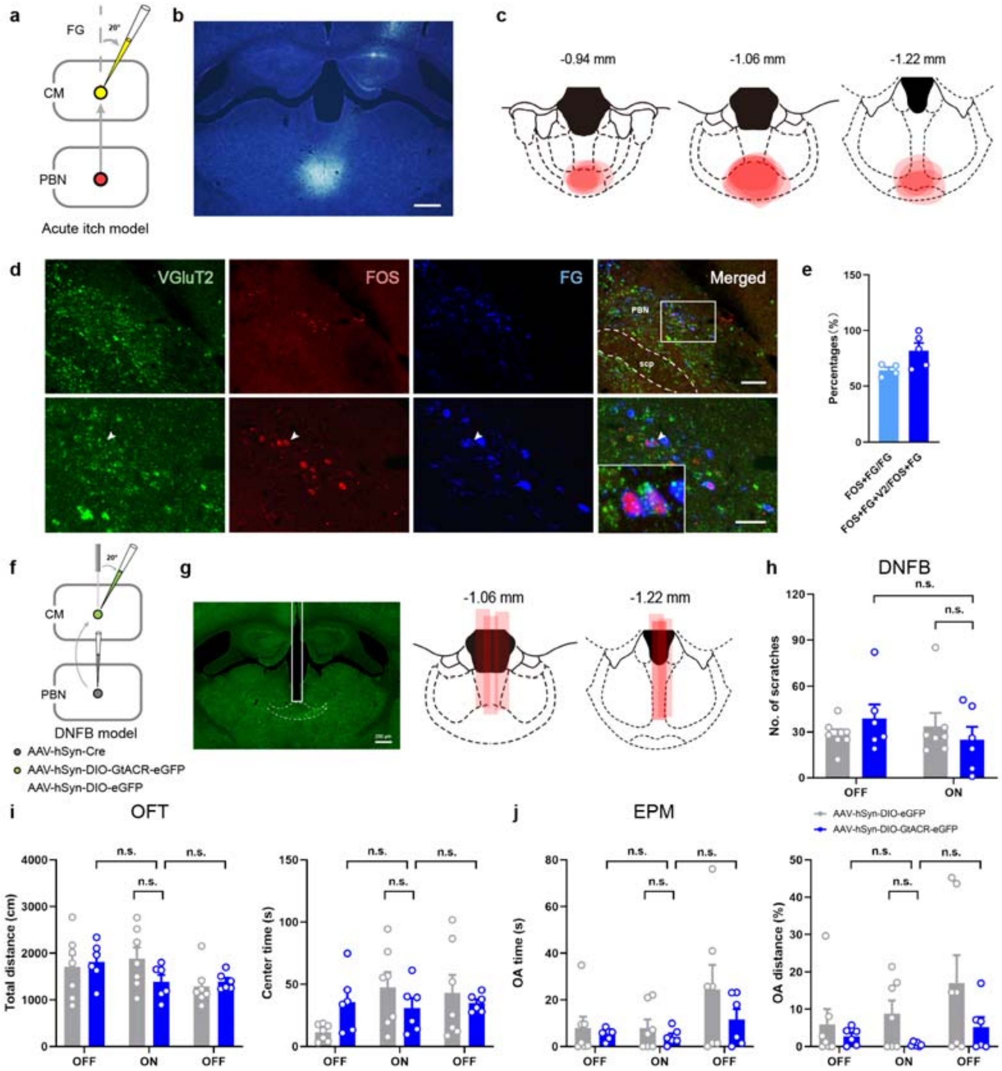

Supplementary Fig. 3 Glutamatergic projections from the PBN sent itch signal to the CM. a, Injection of retrograde tracer Fluoro-gold (FG) into the CM with mice performed with acute itch model before sacrifice. **b**, Representative image of FG injection site (n=5). Scale bars=500 μm. **c**, Rostral-caudal distribution of the FG injection site (red shadow). **d**, Representative images of FOS immunoreactivity in VGluT2 mRNA positive and FG-labeled neurons. White square is enlarged and VGluT2 mRNA(green)-/FOS (red)-/FG (blue)- triple-labeled neurons pointed with white arrows are displayed as insert image. Scale bars=100 μm (upper), 40 μm (lower). **e**, Quantitative analysis showing the percentage of FOS/FG double-labeled neurons in CM-projecting neurons, and VGluT2

mRNA/FOS/FG triple-labeled neurons in itch-activated CM-projecting neurons in the PBN (*n*=4). **f-g**, Schematic diagram and the optic fiber implantation sites (red shadow) in specific optogenetic inhibition of the PBN-CM pathway in mice performed with DNFB model. Scale bars=200 μm. **h**, The total number of scratches summarized during light on (15 min) and light off (15 min) phases in mice from DNFB group (interaction: P=0.2240, treatment: P=0.8998, GtACR (light-off vs. light-on): P=0.8027, light-on (GtACR vs. eGFP): P=0.9676; n=7 for eGFP group, *n*=6 for GtACR group). **i**, Summary data of mice behavior in OFT during light on and light off phases (Total distance: interaction: P=0.2111, treatment: P=0.5507, GtACR (first light-off vs. light-on): P=0.8924, GtACR (light-on vs. last light-off): P > 0.9999, light-on (GtACR vs. eGFP): P=0.7014; Center time: interaction: P=0.0960, treatment: P=0.9771, GtACR (first light-off vs. light-on): P > 0.9999, GtACR (light-on vs. last light-off): P > 0.9999, light-on (GtACR vs. eGFP): P=0.9784; n=7 for eGFP group, *n*=6 for GtACR group). **j**, Summary data of mice behavior in EPM during light on and light off phases (OA time: interaction: P=0.6194, treatment: P=0.1690, GtACR (first light-off vs. light-on): P > 0.9999, GtACR (light-on vs. last light-off): P=0.9992, light-on (GtACR vs. eGFP): P > 0.9999; OA distance: interaction: P=0.6017, treatment: P=0.0314, GtACR (first light-off vs. light-on): P > 0.9999, GtACR (light-on vs. last light-off): P > 0.9999, light-on (GtACR vs. eGFP): P=0.9452; n=7 for eGFP group, *n*=6 for GtACR group). n.s.: no significance. Data are presented as mean ± S.E.M. Two-way ANOVA with Tukey's multiple comparison tests were used for **h-j**. CM: central medial thalamic nucleus; EPM: elevated plus maze; OFT: open filed test; PBN: parabrachial nucleus; scp: superior cerebellar peduncle.

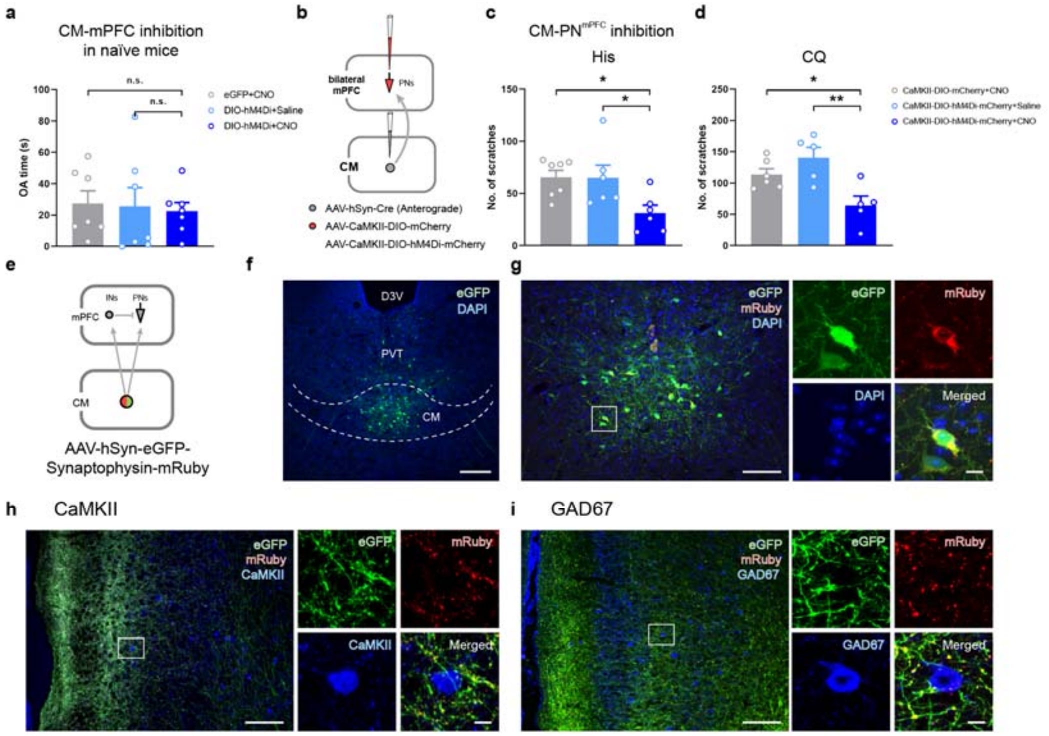

Supplementary Fig. 4 Projections from the CM to the glutamatergic/GABAergic neurons in the mPFC. **a**, Summary data of OA time in naïve mice when inhibiting the CM-mPFC pathway (F(2,18)=0.07931, P=0.9241, CNO (eGFP vs. hM4Di): P=0.9183, hM4Di (Saline vs. CNO): P=0.9672; *n*=7 per group). **b**, Schematic indicating the virus strategy for the CM-PN^{mPFC} pathway-specific inhibition. **c-d**, CM-PN^{mPFC} pathway-specific inhibition significantly relieved the scratching behavior of mice in His- and CQ-induced acute itch models (His: F(2,16)=4.777, P=0.0236, CNO (mCherry vs. hM4Di): P=0.0354, hM4Di (Saline vs. CNO): P=0.046; n=7, 6, 6 per group; CQ: F(2,13)=7.942, P=0.0056, CNO (mCherry vs. hM4Di): P=0.0496, hM4Di (Saline vs. CNO): P=0.0046; n=6, 5, 5 per group). n.s.: no significance, $*P < 0.05$, $**P < 0.01$. Data are presented as mean ± S.E.M. One-way ANOVA with Tukey's multiple comparison tests were used for **a, c-d**. **e**, Schematic diagram of the AAV-hSyn-eGFP-Synaptophysin-mRuby injection into the CM. **f**, Representative images of virus injection site in the CM. Scale bar=200 μm. **g**, Detailed virus injection site in the CM. The white square is further enlarged as the images on the right. Scale bars=100 μm (left), 10 μm (right). **h**, Distributions of eGFP-labeled fibers from the CM to pyramidal neurons (PNs) labeled by CaMKII in the mPFC. The triple staining of eGFP-labeled fibers (green), mRuby labeled synaptophysin-immunoreactive (ir) terminals (red) and CaMKII neurons (blue) in the layer II/III

of the mPFC (right). Scale bar=100 μm (left), 10 μm (right). **i**, By using the same protocol as in **h**, the triple staining of eGFP-labeled fibers (green), synaptophysin-ir terminals (red) and pan GABAergic interneurons (INs) marker GAD67 (blue) were observed in the mPFC. Three mice were used in f, h and i, respectively. The experiment was repeated three times with similar results in each animal. Scale bar=100 μm (left), 10 μm (right).

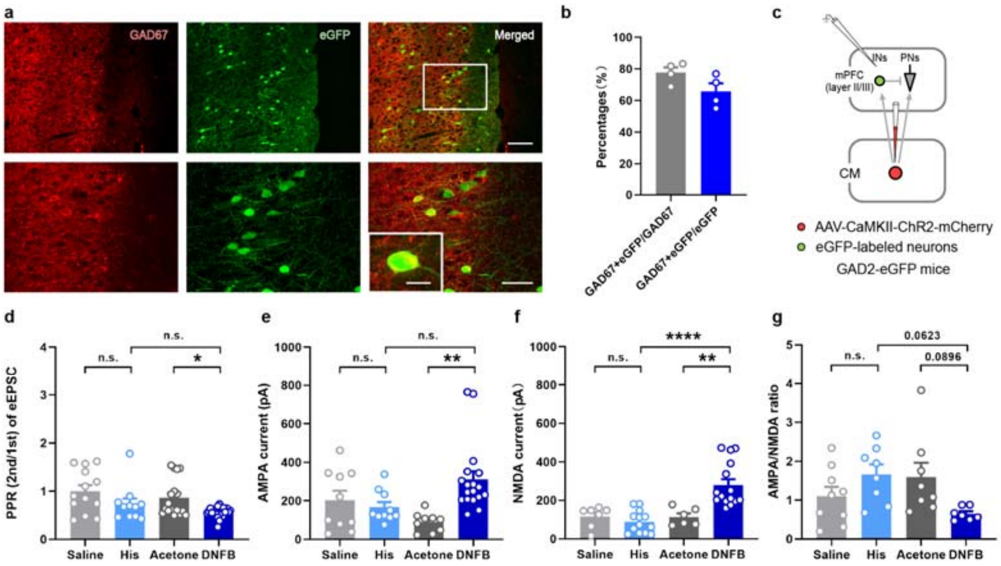

Supplementary Fig. 5 Electrophysiological changes in GAD2-eGFP mice of acute and chronic itch models. a, Verification of the effectiveness of the AAV2/9-mdlx-eGFP virus. The pan GABAergic interneurons (INs) marker GAD67 (red)/mdlx-eGFP (green) double-labeled neurons were observed in lamina II/III in the mPFC (n=3. The experiment was repeated three times with similar results in each animal). Scale bar=100 μm (upper), 40 μm (lower), 10 μm (inner box). **b**, Percentages of GAD67/eGFP double-labeled neurons in GAD67 and eGFP-labeled neurons, respectively (*n*=4). **c**, Schematic diagram of the AAV-CaMKII-ChR2-mCherry injection into the CM of the GAD2-eGFP mice. **d**, Summary data of INs-mediated PPR from the GAD2-eGFP mice in Saline, His, Acetone and DNFB groups ($F(3,62)=6.261$, $P=0.0009$, Saline vs. His: $P=0.1872$, Ace vs. DNFB: $P=0.02$, His vs. DNFB: $P=0.4486$; Saline: $n=13$ from 3 mice; His: $n=11$ from 4 mice; Ace:

n=17 from 3 mice; DNFB: *n*=25 from 4 mice). **e-g**, Summary data of INs-mediated AMPA current, NMDA current and AMPA/NMDA ratio in acute and chronic itch models (AMPA current: F(3,43)=5.784, P=0.0021, Saline vs. His: P=0.9366, Ace vs. DNFB: P=0.0017, His vs. DNFB: P=0.0539; n=10, 10, 9, 18 neurons per group. NMDA current: F(3,37)=14.82, P＜0.0001, Saline vs. His: P=0.8909, Ace vs. DNFB: P=0.0011, His vs. DNFB: P＜0.0001; n=7, 13, 6, 15 neurons per group. AMPA/NMDA ratio: F(3,28)=3.031, P=0.0458, Saline vs. His: P=0.4179, Ace vs. DNFB: P=0.0896, His vs. DNFB: P=0.0623; Saline: *n*=9 from 4 mice; His: *n*=8 from 5 mice; Ace: *n*=8 from 4 mice; DNFB: *n*=7 from 4 mice). n.s.: no significance, *P < 0.05, **P < 0.01, ****P < 0.0001. Data are presented as mean ± S.E.M. One-way ANOVA with Tukey's multiple comparison tests for **d-g**.

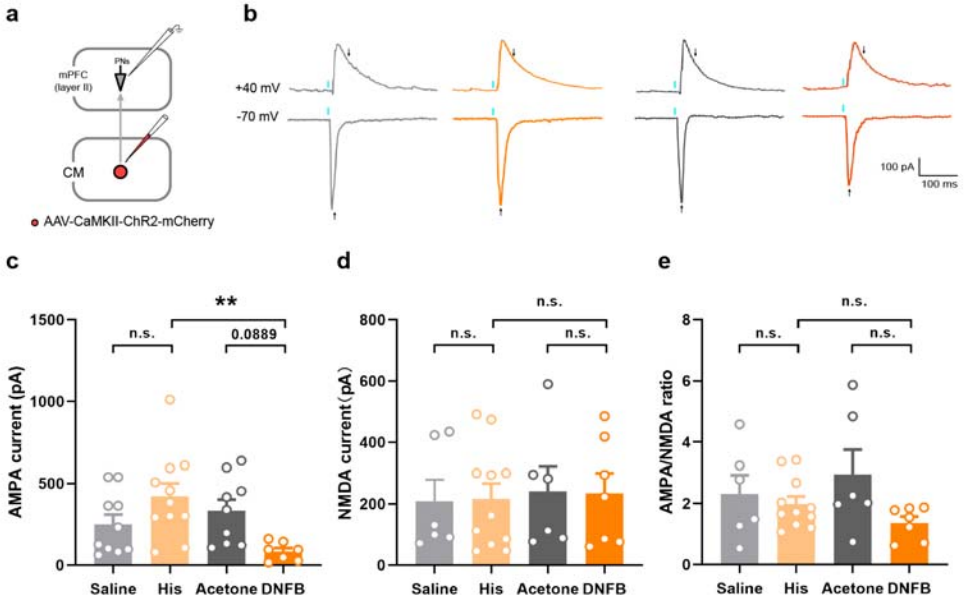

Supplementary Fig. 6 PNs-mediated AMPA and NMDA currents in acute and chronic itch models. a, Schematics of the virus injection and patch configuration of the PNs in the mPFC. **b,** Representative traces of blue light evoked AMPA (voltage clamped at -70 mV) and NMDA currents (voltage clamped at 40 mV). Black arrows indicate the time points of current amplitude used for further analysis. **c**, Summary data of PNs-mediated AMPA current in acute and chronic itch groups

(F(3,33)=4.226, P=0.0124, Saline vs. His: P=0.235, Ace vs. DNFB: P=0.0889, His vs. DNFB: P=0.0081; n=10, 11, 9, 7 neurons per group). **d-e**, NMDA current and AMPA/NMDA ratio remained unchanged in DNFB group when compared with that in His and Acetone groups (NMDA: F(3,26)=0.05006, P=0.9849, Saline vs. His: P=0.9997, Ace vs. DNFB: P=0.9999, His vs. DNFB: P=0.9964; n=6, 11, 6, 7 neurons per group; AMPA/NMDA ratio: F(3,26)=1.890, P=0.1560, Saline vs. His: P=0.9512, Ace vs. DNFB: P=0.1189, His vs. DNFB: P=0.7254; Saline: *n*=6 from 4 mice; His: *n*=11 from 5 mice; Ace: *n*=6 from 5 mice; DNFB: *n*=7 from 6 mice). n.s.: no significance, **P < 0.01, Data are presented as mean ± S.E.M. One-way ANOVA with Tukey's multiple comparison tests for **c-e**.

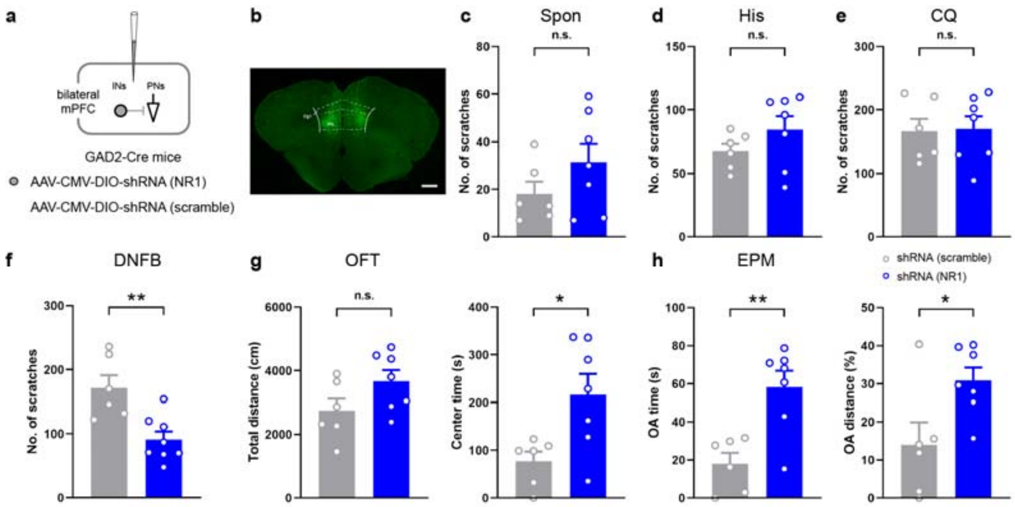

Supplementary Fig. 7 Itch and anxiety-like behavior in mice with specific NR1-knockdown in IN^{mPFC}. a, Specific knockdown of NR1 subunit of NMDA receptor on GABAergic interneurons in the mPFC by injecting AAV-CMV-DIO-shRNA (NR1) into bilateral mPFC. **b**, Representative image of virus injection sites in bilateral mPFC (repeated in 28 successfully-injected mice). Scale bar=500 µm. **c-e**, Summary data of scratching behavior in spontaneous and acute itch groups (Spon: shRNA (scramble) vs. shRNA (NR1): P=0.1973; *n*=6, 7 per group; His: shRNA (scramble) vs. shRNA

(NR1): P=0.1542; *n*=6, 8 per group; CQ: shRNA (scramble) vs. shRNA (NR1): P=0.927; *n*=6, 8 per group). **f**, Summary data of scratching behavior of mice performed with DNFB model (shRNA (scramble) vs. shRNA (NR1): P=0.0033; *n*=6, 8 per group). **g**, NR1 subunit knockdown of the IN^{mPFC} significantly increased the center time of mice in DNFB model without affecting the total distance in OFT (Total distance: shRNA (scramble) vs. shRNA (NR1): P=0.0551; Center time: shRNA (scramble) vs. shRNA (NR1): P=0.0181; *n*=6, 7 per group). **h**, NR1 subunit knockdown of IN^{mPFC} significantly increased the OA time and OA distance in EPM (*n*=6-7) (OA time: shRNA (scramble) vs. shRNA (NR1): P=0.0028; OA distance: shRNA (scramble) vs. shRNA (NR1): P=0.0258; *n*=6, 7 per group). n.s.: not significant, *P < 0.05, **P < 0.01. Data are presented as mean ± S.E.M. Two-tailed, unpaired, Student's t-test for **c**-**h.** Cg1：cingulate cortex, area 1; PrL: prelimbic cortex.

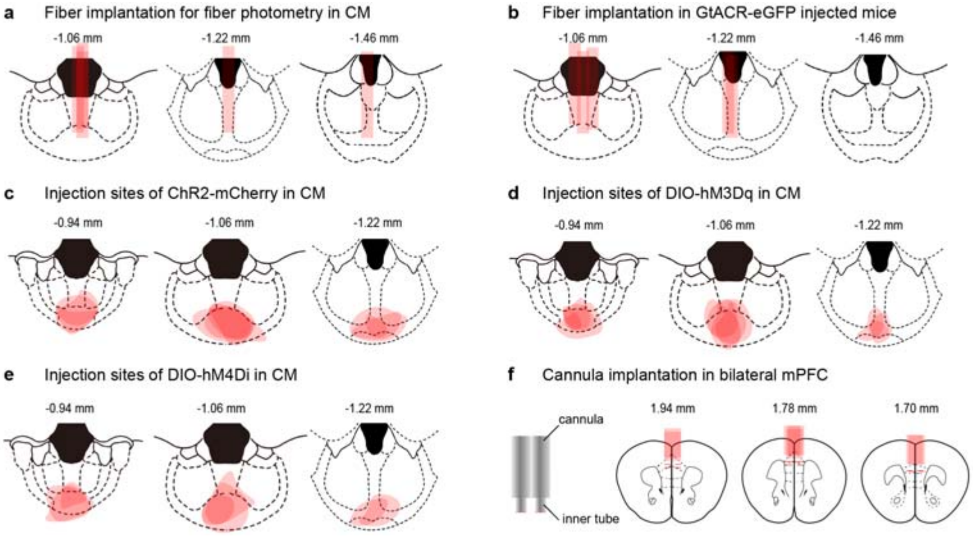

Supplementary Fig. 8 Fiber implantation and virus injection sites. a, Schematic plot of fiber implantation for fiber photometry in the CM. **b**, Schematic plot of fiber implantation in GtACR-eGFP injected mice. **c-e**, Schematic plot of injection sites of ChR2-mCherry, DIO-hM3Dq and DIO-hM4Di viruses in the CM, respectively. **f**, Schematic plot of both cannula and cannula implantation in bilateral mPFC. The inner tube is 0.5 mm longer than the cannula, which is indicated by two red lines at the bottom. Red shadow area represents optic fiber or cannula implantation sites, as well as the virus injection sites in mice.

Supplementary table 1. Viruses and drugs used for anatomical or behavioral purposes

	**Experimental purposes**	**Viruses/Drugs**	**Location/ Target nuclei**	**Volume**	**Serotype**	**Serial Number**
**Morphological**	FOS expression	His/CQ/Saline	nape	15 µl		
**experiments**		Acetone/DNFB	nape			
	Retrograde	FG	right mPFC/CM	0.04 µl		
	tracing	488-retrobeads	Bilateral mPFC	150 nl		
	Anterograde	AAV-hSyn-eGFP-Synaptophysin-mRuby	right PBN	150 nl	AAV2/9	PT-0937
	tracing	AAV-cFos-eYFP	right PBN	150 nl	AAV2/9	PT-0129
		AAV-hSyn-Cre-mCherry	right PBN	150 nl	AAV2/9	PT-0407
**Behavioral**	Baseline	His/CQ/Saline	nape	15 µl		
**experiments**		Acetone/DNFB	nape			
	Fiber photometry	AAV-CaMKII-GCaMP6m	CM	150 nl	AAV2/9	PT-0111
		AAV-hSyn-Cre	right PBN	150 nl	AAV2/1	PT-0241
		AAV-hSyn-DIO-GCaMP7s	CM	150 nl	AAV2/9	PT-1421
	Pharmacological	AAV-CMVbGlobin-Cre-mCherry	Right mPFC	150 nl	AAV2/R	T1108*
	manipulation	AAV-hSyn-DIO-hM3Dq-eGFP	CM	150 nl	AAV2/9	PT-0152
		AAV-hSyn-DIO-hM4Di-eGFP	CM	150 nl	AAV2/9	PT-0153
	Optogenetic	AAV-hSyn-DIO-GtACR-eGFP	CM	150 nl	AAV2/9	PT-1368
	manipulation	AAV-hSyn-DIO-eGFP	CM	150 nl	AAV2/9	PT-1103
		AAV-mdlx-DIO-hM3Dq-eGFP	Bilateral mPFC	150 nl	AAV2/9	PT-2654
	NR1 knockdown	AAV-CMV-DIO-(EGFP-U6)-shRNA (NR1)	Bilateral mPFC	150 nl	AAV2/9	PT-5878
		AAV-CMV-DIO-(EGFP-U6)-shRNA(scramble)	Bilateral mPFC	150 nl	AAV2/9	PT-2644
**Electrophysiological**		AAV-mdlx-eGFP	Bilateral mPFC	150 nl	AAV2/9	PT-2651
**experiments**		AAV-CaMKII-hChR2(H134R)-mCherry	CM	150 nl	AAV2/9	PT-0005

*: AAV-CMVbGlobin-Cre-mCherry were provided by Shanghai Taitool Bioscience Co. Ltd. Other viruses were provided by Wuhan Brain VTA Co. Ltd.

Supplementary table 2. Antisera used for DAB reaction, FISH staining and immunofluorescent staining

Groups	Primary antisera	Secondary antisera	Tertiary antisera
**DAB reaction**	Mouse anti-FOS<br>(1:500, ab11959, Abcam, MA, UK)	Biotin-donkey anti-mouse<br>(1:500, AP192B, Merck Millipore, CA, USA)	ABCkit<br>(1:200, PK-6101, Vectorlabs, CA, USA)
**GFAP/NeuN**	Rabbit anti-GFAP<br>(1:500, ab7260, Abcam)<br>Mouse anti-NeuN<br>(1:500, mab377, Merck Millipore)	Alexa 488 donkey anti-rabbit<br>(1:500, A21206, Invitrogen, CA, USA)<br>Alexa 594 donkey anti-mouse<br>(1:500, A21203, Invitrogen)	
**FOS/eYFP/DAPI**	Mouse anti-FOS<br>(1:500, ab11959, Abcam)	Alexa 594 donkey anti-mouse<br>(1:500, A21203, Invitrogen)	DAPI<br>(1:500, sc-3598, Santacruz, TX, USA)
**VGluT2/FG/FOS**	VGluT2 riboprobes	anti-digoxigenin sheep antibody<br>(1:1500, 11-207-733-910, Roche Diagnostic, Basel, Switzerland)<br>Rabbit anti-FG antibody<br>(1:1000, AB153-I, Merck Millipore)<br>mouse anti-FOS antibody<br>(1:500, ab11959, Abcam)	FITC-Avidin<br>(1:500, A-2001, Vectorlabs)<br>Alexa 647-donkey anti-rabbit<br>(1:500, A31573, Invitrogen)<br>Alexa 594-donkey anti-mouse<br>(1:500, A21203, Invitrogen)
**eGFP/mRuby/FG**	Rabbit anti-FG antibody<br>(1:1000, AB153-I, Merck Millipore)	Alexa 647-donkey anti-rabbit<br>(1:500, A31573, Invitrogen)	
**eGFP/mRuby/CaMKII**	Mouse anti-CaMKII antibody<br>(1:500, ab22609, Abcam)	Alexa 647-donkey anti-mouse<br>(1:500, A31571, Invitrogen)	
**eGFP/mRuby/GAD67**	Mouse anti-GAD67 antibody<br>(1:500, MAB5406, Merck Millipore)	Alexa 647-donkey anti-mouse<br>(1:500, A31571, Invitrogen)	
**Biocytin**	647-avidin<br>(1:500, S21374, Invitrogen)		

Supplementary table 3. The company names and serial number of drugs used in experiments

Chemicals	Company names	Serial Number
His	Sigma, MO, USA	H7250
CQ	Sigma, MO, USA	C6628
DAB	Sigma, MO, USA	D12384
1-fluoro-2,4-dinitrobenzene (DNFB)	Sigma, MO, USA	D1529
Clozapine N-oxide (CNO)	Sigma, MO, USA	C8032
488-retrobeads	Lumafluor, New York, NY, USA	Cas:78G180
tetrodotoxin (TTX)	Enzo Life Sciences, Farmingdale, NY, USA	BML-NA120
4-aminopyridine (4-AP)	Sigma, MO, USA	A78403
AP-5	Sigma, MO, USA	A6553
Picrotoxin (PTX)	Sigma, MO, USA	R284556
CNQX	Sigma, MO, USA	C127
Biocytin	Sigma, MO, USA	B4261
Lanicemine	Sigma, MO, USA	SML0635
