# Supplementary material for: Central medial thalamic nucleus dynamically participates in acute itch sensation and chronic itch-induced anxiety-like behavior in male mice
Source: Nat Commun. 2023 May 3;14:2539. doi: 10.1038/s41467-023-38264-4 (PMC10156671; doi:10.1038/s41467-023-38264-4)
Supplement: Supplementary file 3 — Reporting Summary [file 41467_2023_38264_MOESM3_ESM.pdf]

nature portfolio

Corresponding author(s): Yu-Lin Dong
Yun-Qing Li

Last updated by author(s): Mar 10, 2023

Reporting Summary

Nature Portfolio wishes to improve the reproducibility of the work that we publish. This form provides structure for consistency and transparency in reporting. For further information on Nature Portfolio policies, see our Editorial Policies and the Editorial Policy Checklist.

Statistics

For all statistical analyses, confirm that the following items are present in the figure legend, table legend, main text, or Methods section.

n/a	Confirmed	
☐	☒	The exact sample size (*n*) for each experimental group/condition, given as a discrete number and unit of measurement
☐	☒	A statement on whether measurements were taken from distinct samples or whether the same sample was measured repeatedly
☐	☒	The statistical test(s) used AND whether they are one- or two-sided<br>*Only common tests should be described solely by name; describe more complex techniques in the Methods section.*
☐	☒	A description of all covariates tested
☐	☒	A description of any assumptions or corrections, such as tests of normality and adjustment for multiple comparisons
☐	☒	A full description of the statistical parameters including central tendency (e.g. means) or other basic estimates (e.g. regression coefficient) AND variation (e.g. standard deviation) or associated estimates of uncertainty (e.g. confidence intervals)
☐	☒	For null hypothesis testing, the test statistic (e.g. *F*, *t*, *r*) with confidence intervals, effect sizes, degrees of freedom and *P* value noted<br>*Give P values as exact values whenever suitable.*
☒	☐	For Bayesian analysis, information on the choice of priors and Markov chain Monte Carlo settings
☒	☐	For hierarchical and complex designs, identification of the appropriate level for tests and full reporting of outcomes
☒	☐	Estimates of effect sizes (e.g. Cohen's *d*, Pearson's *r*), indicating how they were calculated

Our web collection on statistics for biologists contains articles on many of the points above.

Software and code

Policy information about availability of computer code

Data collection	Confocal imaging (Olympus,SystemFV1000). Clampex software (v.10.02, Axon Instruments) for whole-cell patch-clamp recordings;Video system from Shanghai Mobile Datum was used in open field test and elevated plus maze. Fiber photometry system (InterStudioMultiColorEVAL15,InperLtd).
Data analysis	Imaging data were analyzed with FV10-ASW 4.2 Viewer and Image J 1.53e. Electrophysiology data were analyzed by Mini Analysis (v.6.0, Synaptosoft Inc.) and Clampfit(v.10.7, Axon Instruments). Fiber photometry data by InperDataProcess V.0.7.2. Data were graphed and analyzed using Graphpad Prism (v.8.4.2) and OriginPro (v.9.8)

For manuscripts utilizing custom algorithms or software that are central to the research but not yet described in published literature, software must be made available to editors and reviewers. We strongly encourage code deposition in a community repository (e.g. GitHub). See the Nature Portfolio guidelines for submitting code & software for further information.

Data

Policy information about availability of data

All manuscripts must include a data availability statement. This statement should provide the following information, where applicable:

- Accession codes, unique identifiers, or web links for publicly available datasets
- A description of any restrictions on data availability
- For clinical datasets or third party data, please ensure that the statement adheres to our policy

The datasets generated during and/or analysed during the current study are available from the corresponding author on reasonable request.

Human research participants

Policy information about studies involving human research participants and Sex and Gender in Research.

Reporting on sex and gender	*Use the terms sex (biological attribute) and gender (shaped by social and cultural circumstances) carefully in order to avoid confusing both terms. Indicate if findings apply to only one sex or gender; describe whether sex and gender were considered in study design whether sex and/or gender was determined based on self-reporting or assigned and methods used. Provide in the source data disaggregated sex and gender data where this information has been collected, and consent has been obtained for sharing of individual-level data; provide overall numbers in this Reporting Summary. Please state if this information has not been collected. Report sex- and gender-based analyses where performed, justify reasons for lack of sex- and gender-based analysis.*
Population characteristics	*Describe the covariate-relevant population characteristics of the human research participants (e.g. age, genotypic information, past and current diagnosis and treatment categories). If you filled out the behavioural & social sciences study design questions and have nothing to add here, write "See above."*
Recruitment	*Describe how participants were recruited. Outline any potential self-selection bias or other biases that may be present and how these are likely to impact results.*
Ethics oversight	*Identify the organization(s) that approved the study protocol.*

Note that full information on the approval of the study protocol must also be provided in the manuscript.

Field-specific reporting

Please select the one below that is the best fit for your research. If you are not sure, read the appropriate sections before making your selection.

☒ Life sciences ☐ Behavioural & social sciences ☐ Ecological, evolutionary & environmental sciences

For a reference copy of the document with all sections, see nature.com/documents/nr-reporting-summary-flat.pdf

Life sciences study design

All studies must disclose on these points even when the disclosure is negative.

Sample size	No statistical methods were used to pre-determine sample sizes. Sample sizes adopted in this study were sufficient for detecting effects. The number of animals in each group were determined according to previous studies (PMID: 32841213; PMID: 33449512).
Data exclusions	In behavior tests, data were excluded when injections sites missed the target nuclei. In electrophysiological test, data were excluded when the resting membrane potential of neurons were more positive than -50 mV. In tracer tracing studies, data from mice with apparent injection sites spreading to other nuclei were removed.
Replication	Each experiment was successfully repeated for at least two times to verify the reproducibility of experimental findings.
Randomization	The mice were randomized to put into separate groups for allocation.
Blinding	The investigators were blinded to group allocation during data collection and blinded to the treatment of mice to which mice were subjected when assessing behavioral and neurophysiological test.

Reporting for specific materials, systems and methods

We require information from authors about some types of materials, experimental systems and methods used in many studies. Here, indicate whether each material, system or method listed is relevant to your study. If you are not sure if a list item applies to your research, read the appropriate section before selecting a response.

Materials & experimental systems

n/a	Involved in the study
☐	☒ Antibodies
☒	☐ Eukaryotic cell lines
☒	☐ Palaeontology and archaeology
☐	☒ Animals and other organisms
☒	☐ Clinical data
☒	☐ Dual use research of concern

Methods

n/a	Involved in the study
☒	☐ ChIP-seq
☒	☐ Flow cytometry
☒	☐ MRI-based neuroimaging

Antibodies

Antibodies used

Primary antibody used:
Mouse monoclonal anti-FOS (1:500, Cat. # ab11959, Abcam, MA, UK)
Rabbit polyclonal anti-GFAP (1:500, Cat. # ab7260, Abcam)
Mouse monoclonal anti-NeuN (1:500, Cat. # mab377, Merck Millipore)
Rabbit polyclonal anti-FG antibody (1:1000, Cat. # AB153-I, Merck Millipore)
Mouse monoclonal anti-CaMKII antibody (1:500, Cat. # ab22609, Abcam)
Mouse monoclonal anti-GAD67 antibody (1:500, Cat. # MAB5406, Merck Millipore)
Anti-digoxigenin polyclonal sheep antibody (1:1500, Cat. # 11-207-733-910, Roche Diagnostic, Basel, Switzerland)
Secondary antibody used:
Biotin-donkey anti-mouse (1:500, Cat. # AP192B, Merck Millipore, CA, USA)
Alexa 488 donkey anti-rabbit (1:500, Cat. # A21206, Invitrogen, CA, USA)
Alexa 594 donkey anti-mouse (1:500, Cat. # A21203, Invitrogen)
Alexa 647-donkey anti-rabbit (1:500, Cat. # A31573, Invitrogen)
Alexa 647-donkey anti-mouse (1:500, Cat. # A31571, Invitrogen)
647-avidin (1:500, Cat. # S21374, Invitrogen)
FITC-Avidin (1:500, Cat. # A-2001, Vectorlabs)

Validation

The specificity and application of all the primary antibodies are validated by companies:
Mouse anti-FOS
Validation: https://www.abcam.cn/fos-b-antibody-83b1138-ab11959.html
Rabbit anti-GFAP
Validation: https://www.abcam.cn/gfap-antibody-ab7260.html
Mouse anti-NeuN
Validation: https://www.sigmaaldrich.cn/CN/zh/product/mm/mab377
Rabbit anti-FG antibody
Validation: https://www.sigmaaldrich.cn/CN/zh/product/mm/ab153i
Mouse anti-CaMKII antibody
Validation: https://www.abcam.cn/camkii-antibody-6g9-ab22609.html
Mouse anti-GAD67 antibody
Validation: https://www.sigmaaldrich.cn/CN/zh/product/mm/mab5406
Anti-digoxigenin sheep antibody
Validation: https://www.sigmaaldrich.cn/CN/zh/product/roche/11207733910

Animals and other research organisms

Policy information about studies involving animals; ARRIVE guidelines recommended for reporting animal research, and Sex and Gender in Research

Laboratory animals

Mus musculus, C57Bl/6 mice, GAD2-Cre mice and GAD2-eGFP mice; male (8-12 wk, 20-35 g). All animals used were housed in a 12 h light/dark cycle with food and water given ad libitum. Ambient temperature (18_26?) and humidity (40-70%) have been controlled.

Wild animals

The study did not involve wild animals.

Reporting on sex

A total number of 417 C57Bl/6 mice, 28 GAD2-Cre male mice and 35 GAD2-eGFP male mice ware used in this experiment. Only male mice were used because of their steady hormone level, and all previously published paper from our group.

Field-collected samples

The study did not involve samples collected from the field.

Ethics oversight

Protocols were in accordance with the Animal Care and Use Committees at The Fourth Military Medical University (Xi'an, China)

Note that full information on the approval of the study protocol must also be provided in the manuscript.
